# Insights into the Role of PPARβ/δ in NAFLD

**DOI:** 10.3390/ijms19071893

**Published:** 2018-06-27

**Authors:** Jiapeng Chen, Alexandra Montagner, Nguan Soon Tan, Walter Wahli

**Affiliations:** 1 Lee Kong Chian School of Medicine, Nanyang Technological University, 11 Mandalay Road, Singapore 308232, Singapore; jchen025@e.ntu.edu.sg (J.C.); nstan@ntu.edu.sg (N.S.T.); 2 School of Biological Sciences, Nanyang Technological University, 60 Nanyang Drive, Singapore 637551, Singapore; 3 ToxAlim, Research Center in Food Toxicology, National Institute for Agricultural Research (INRA), 180 Chemin de Tournefeuille, 31300 Toulouse, France; alexandra.montagner@inserm.fr; 4 Institut National de La Santé et de La Recherche Médicale (INSERM), UMR1048, Institute of Metabolic and Cardiovascular Diseases, 31027 Toulouse, France; 5 KK Research Centre, KK Women’s and Children Hospital, 100 Bukit Timah Road, Singapore 229899, Singapore; 6 Institute of Molecular and Cell Biology, Agency for Science Technology & Research, 61 Biopolis Drive, Proteos, Singapore 138673, Singapore; 7 Center for Integrative Genomics, University of Lausanne, Génopode, CH-1015 Lausanne, Switzerland

**Keywords:** PPARβ/δ, NAFLD, NASH, steatosis, liver, lipid metabolism

## Abstract

Non-alcoholic fatty liver disease (NAFLD) is a major health issue in developed countries. Although usually associated with obesity, NAFLD is also diagnosed in individuals with low body mass index (BMI) values, especially in Asia. NAFLD can progress from steatosis to non-alcoholic steatohepatitis (NASH), which is characterized by liver damage and inflammation, leading to cirrhosis and hepatocellular carcinoma (HCC). NAFLD development can be induced by lipid metabolism alterations; imbalances of pro- and anti-inflammatory molecules; and changes in various other factors, such as gut nutrient-derived signals and adipokines. Obesity-related metabolic disorders may be improved by activation of the nuclear receptor peroxisome proliferator-activated receptor (PPAR)β/δ, which is involved in metabolic processes and other functions. This review is focused on research findings related to PPARβ/δ-mediated regulation of hepatic lipid and glucose metabolism and NAFLD development. It also discusses the potential use of pharmacological PPARβ/δ activation for NAFLD treatment.

## 1. Introduction

Non-alcoholic fatty liver disease (NAFLD) is an inclusive term describing a broad range of chronic liver pathologies [1]. During the development of this chronic condition, several potentially pathogenic mediators are crucially involved [2]. Risk factors for NAFLD include obesity, insulin resistance, and other features of metabolic syndrome. Steatosis is the initial benign stage, characterized by lipid accumulation in hepatocytes due to impaired triglyceride synthesis and export, and/or reduced fatty acid beta-oxidation. Patients with steatosis may progress to non-alcoholic steatohepatitis (NASH), a more severe form of NAFLD that involves hepatocellular injury and liver inflammation—both drivers of hepatic fibrosis [3]. NASH can lead to more deleterious conditions, such as cirrhosis and hepatocellular carcinoma (HCC) [4]. NASH is rapidly becoming a leading cause of end-stage liver disease and hepatocellular carcinoma, both of which are indications for liver transplantation [5].

As obesity rates have risen, NAFLD has become the most common chronic liver disease in humans and is considered an epidemic disease that constitutes a major global health issue. NAFLD affects 70% of type 2 diabetes patients, and even a greater proportion of obese diabetic individuals [6,7]. Astonishingly, NAFLD affects nearly 30% of the general population worldwide [8,9,10] and has potentially serious sequelae [11]. Although steatosis is considered a relatively benign condition, about 30% of patients with steatosis will develop NASH, and 30–40% of patients with NASH will progress to fibrosis and cirrhosis. Among patients with cirrhosis, 4% will develop hepatocellular carcinoma with a 10-year mortality rate of 25% [12,13,14].

Although the majority of affected individuals are asymptomatic, NAFLD can be detected by ultrasound scanning or routine blood testing for elevated plasma levels of the liver enzymes alanine aminotransferase and aspartate aminotransferase, reflecting hepatocyte injury. On the other hand, NASH diagnosis requires a liver biopsy and histological scoring. Individuals who are diabetic or obese, or who suffer from metabolic syndrome, should be suspected as having NAFLD and should be examined accordingly [15,16,17].

Body weight reduction through increased physical activity and dietary improvement can help with NAFLD management and delay disease progression. However, long-term lifestyle changes may be insufficient in many cases [18,19,20]. Notably, there is currently no effective FDA-approved therapy for the prevention and/or treatment of NAFLD development and progression, although several drugs are currently being tested in clinical trials [21]. Pharmacological treatments that target insulin resistance, including metformin and thiazolidinediones (TZDs), have been tested in NAFLD patients and those diagnosed with NASH. These studies have not demonstrated that metformin is effective for NAFLD treatment [21,22]. TZDs reportedly lead to decreased hepatic fat and reduced liver injury; however, TZD discontinuation allows NASH recurrence, and long-term TZD treatment can result in medical complications, such as congestive heart failure, osteoporosis, and weight gain in susceptible patients [23,24]. Thus, other than weight loss, there are currently no effective interventions and therapies for NAFLD treatment [18,19,20,21].

Peroxisome proliferator-activated receptor (PPAR)β/δ is a nuclear receptor that is closely related to PPARγ, which is activated by TZDs, as well as to PPARα, which is targeted by hypolipidemic agents of the fibrate class. PPARβ/δ exerts a variety of metabolic effects and physiological actions [25,26,27,28,29], and PPARβ/δ activation may inhibit and improve obesity-related metabolic disorders. In the present review, we discuss the involvement of PPARβ/δ in NAFLD, and the effects of PPARβ/δ agonists on this pathology.

## 2. Hallmark of NAFLD

### 2.1. Two-Hit Hypothesis

**Figure 1:**
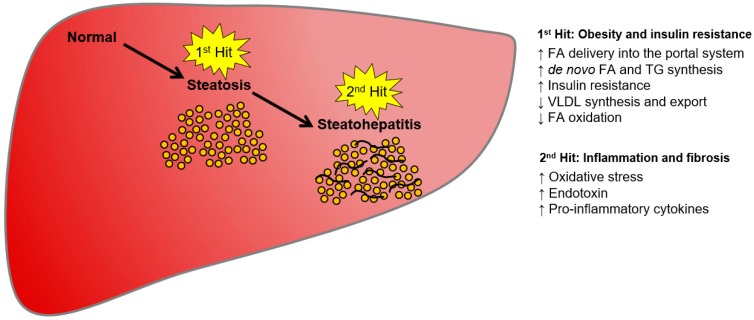
Schematic diagram of the two-hit hypothesis of non-alcoholic fatty liver disease (NAFLD) progression. In the first hit, an imbalance of lipid synthesis, catabolism, and export results in lipid accumulation in liver (steatosis). Obesity and insulin resistance are strongly correlated with liver steatosis. In the second hit, further inflammation processes lead to non-alcoholic steatohepatitis (NASH) and fibrosis, which can evolve into more severe stages, such as cirrhosis and ultimately hepatocellular carcinoma.

It has been proposed that NAFLD pathogenesis is a “two-hit” process (Figure 1) [30,31]. In this hypothesis, the first hit results from triglyceride accumulation in the hepatocyte cytoplasm due to an imbalance in lipid input and output, which is the hallmark of NAFLD [30]. Four mechanisms can contribute to triglyceride accumulation in hepatocytes: (1) upregulated free fatty acid uptake from blood plasma in the context of increased lipolysis from adipose tissue and/or chylomicrons after high-fat diet consumption [32]; (2) high carbohydrate uptake that increases circulating glucose and insulin levels, thus promoting de novo lipogenesis and contributing to triglyceride accumulation in hepatocytes [33,34]; (3) decreased fatty acid mitochondrial oxidation; and (4) reduced hepatic triglyceride secretion via packaging of apolipoprotein B (ApoB) into very low-density lipoprotein (VLDL) particles, promoting triglyceride accumulation in hepatocytes [33,34,35]. Overall, aberrations in any lipid metabolism processes, which may involve a large number of genes, can result in NAFLD development [36].

The second hit in this NAFLD progression model is an imbalance of pro- and anti-inflammatory factors, resulting in increased inflammation, as seen in NASH [30]. Hence, the most critical and challenging step in NAFLD progression is the transition from relatively benign steatosis to the damaged and inflamed liver in NASH. Any strong chronic inflammation will cause fibrosis, thereby contributing to the development of cirrhosis and eventually hepatocellular carcinoma [37].

### 2.2. Multiple Parallel Hit Hypothesis

**Figure 2:**
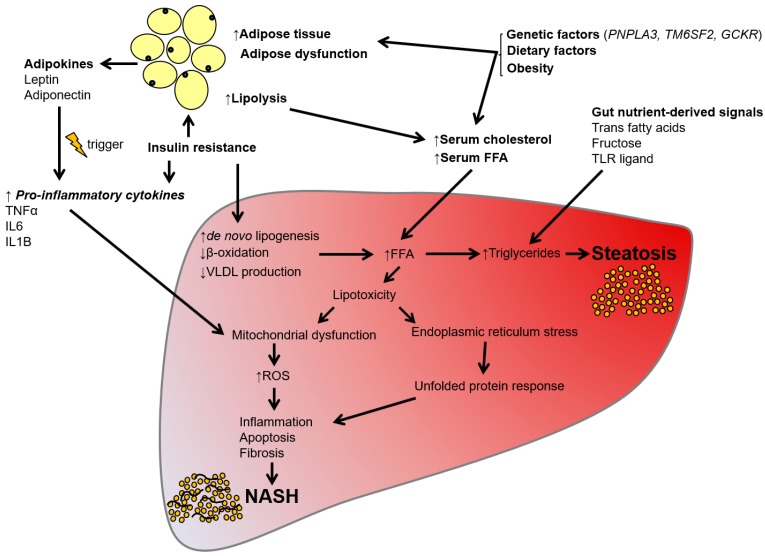
Schematic illustration of the multiple parallel hits hypothesis of NAFLD development. NAFLD develops due to the impaired regulation of several factors, such as gut nutrient-derived signals, adipokines, and certain pro-inflammatory cytokines.

The multiple parallel hit hypothesis considers alterations in the regulation of several factors, including gut nutrient-derived signals, adipokines, and certain pro-inflammatory cytokines (Figure 2) [38]. Insulin resistance leads to alterations of nutrient metabolism and is thus commonly associated with NAFLD development [39]. Elevated levels of inflammatory cytokines, such as interleukin 6 (IL6) and tumor necrosis factor α (TNFα), result in hepatic inflammation [40]. The administration of TNFα antibody into *ob/ob* mice induces steatosis improvement, supporting a role of TNFα in NAFLD progression. Moreover, hepatic steatosis can be induced through primary inflammation in *ob/ob* mice [41]. In humans, inflammation is occasionally observed before steatosis, as seen in patients who have NASH but exhibit lower levels of steatosis [42].

Genome-wide association studies (GWAS) have identified genes that are involved in diseases and that can be targeted for disease treatments. A GWAS of various races found that NAFLD was linked to a polymorphism in the patatin-like phospholipase domain containing 3 (*PNPLA3*) gene [43]. PNPLA3 is a multifunctional enzyme involved in triacylglycerol hydrolysis and acyl-CoA-independent transacylation of acylglycerols [44]. The nonsynonymous rs738409 C/G variant in *PNPLA3* encodes I148M. It is proposed to be the main genetic component of NAFLD and NASH [45]. It reportedly shows the strongest risk effect on NAFLD development, accounting for 5.3% of total variance, and is associated with histological disease severity and NAFLD progression [45,46]. In patients with the single *PNPLA3* nucleotide polymorphism rs738409 G/G, fatty liver progresses directly to NASH [47,48]. Notably, mice with *Pnpla3* deficiency do not develop fatty liver or liver injury [49], and *Pnpla3* knockdown decreases intracellular triglyceride levels in primary hepatocyte cultures [50]. Thus, the function of PNPLA3 in NAFLD warrants further investigation. Interestingly, *Pnpla3* is a downstream target gene of sterol-regulated binding protein 1c (SREBP1c) and can mediate its effect in promoting lipid accumulation. Therefore, PNPLA3 has been suggested as a possible “first hit”, preceding other hits that may affect disease progression [51].

Two other widely studied genetic modifiers of NAFLD are the transmembrane 6 superfamily member 2 (*TM6SF2*) and glucokinase regulator (*GCKR*) genes. TM6SF2 regulates liver fat metabolism, influencing triglyceride secretion and hepatic lipid droplet content [52]. The nonsynonymous rs58542926 variant in *TM6SF2* encodes E167K and is associated with increased liver fat levels [53]. Patients with NAFLD show significantly lower TM6SF2 expression in the liver [54]. With regards to NAFLD risk alleles of *TM6SF2*, the C (Glu167) allele is correlated with higher cardiovascular risk via elevated circulating low-density lipoprotein (LDL)-cholesterol levels [55], and the T (Lys167) allele is associated with NAFLD and NASH [54,56,57]. *GCKR* encodes the glucokinase regulatory protein, which controls the activity and intracellular location of glucokinase, a key enzyme in glucose metabolism [58]. The *GCKR* missense variant rs780094 is significantly associated with histological NAFLD [59,60]. Moreover, *GCKR* mutations reportedly cause maturity-onset diabetes in young individuals with NAFLD risk factors, such as glucose intolerance and insulin resistance [61]. Histological NAFLD is also significantly associated with variants in or near the neurocan (*NCAN)* and lysophospholipase like 1 (*LYPLAL1*), but not protein phosphatase 1 regulatory subunit 3B (*PPP1R3B*) genes [59].

Obesity is another increasingly common global condition that is associated with diseases, including NAFLD, hypertension, type 2 diabetes mellitus, and hyperlipidemia. In fact, hypertension, hypertriglyceridemia, and obesity are predictive risk factors for NAFLD [62]. Over the past decade, visceral obesity has become more common among adults and children worldwide in association with increased consumption of Western-style diets with high fat and fructose contents [63]. Visceral fat accumulation is positively correlated with various organ pathologies, including NAFLD, as well as with insulin resistance in both obese and non-obese individuals. These findings suggest that visceral fat accumulation influences hepatic steatosis, regardless of the degree of obesity [64].

## 3. Peroxisome Proliferator-Activated Receptor β/δ Expression in Liver

**Figure 3:**
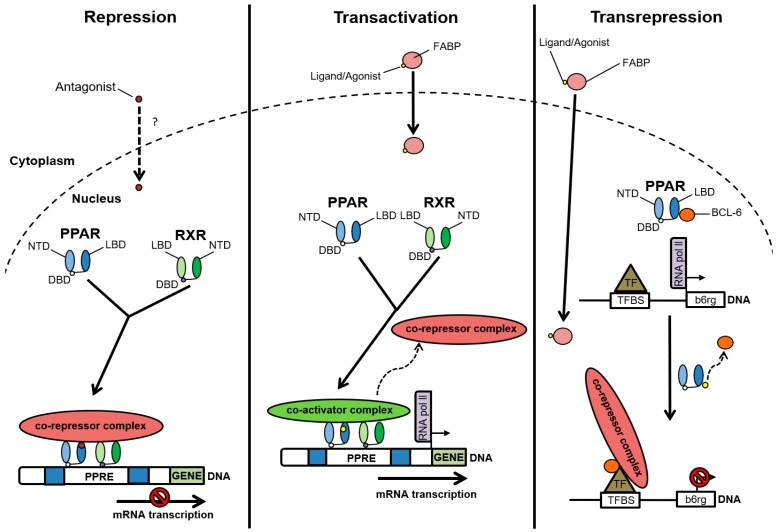
Regulatory mechanisms of gene transcription by peroxisome proliferator-activated receptors (PPARs). Each PPAR structurally comprises an N-terminal domain (NTD), a DNA-binding domain (DBD), and a ligand-binding domain (LBD). In the absence of a ligand or in the presence of an antagonist, the PPAR-RXR heterodimer associates with nuclear receptor co-repressor proteins, leading to repression of PPAR target genes (Repression). Fatty acid-binding protein (FABP) associates with the ligand/agonist to transport it into the cell. Upon ligand binding, a conformational change in PPAR leads to co-repressor dissociation, and co-activators are recruited. The activated PPAR-RXR heterodimer binds the peroxisome proliferator response element (PPRE) and stimulates target gene transcription (Transactivation). In macrophages, endothelial cells, and vascular smooth muscles, in the absence of a PPARβ/δ agonist or ligand, the receptor will scavenge BCL-6 (a PPARβ/δ-associated transcriptional repressor). Once PPARβ/δ neutralizes BCL-6, transcription factors (TFs) bind to TF-binding sites (TFBSs), allows transcription of the genes repressed by BCL-6. However, the binding of a PPARβ/δ ligand to PPARβ/δ will result in BCL-6 dissociation, leading to co-repressor-dependent transrepression of BCL-6 targeted genes, such as *b6rg*, which encodes a sequence-specific transcription repressor (Transrepression). The dashed arrow with a question mark indicates that it is not known how the antagonist is translocated to the cell nucleus. The curvy arrow indicates the dissociation of the co-repressor from the transcription factor.

Peroxisome proliferator-activated receptors (PPARs) belong to the nuclear hormone receptor superfamily, which comprises ligand-activated transcription factors. PPARs play important roles in regulating genes involved in fatty acid uptake and oxidation, lipid and carbohydrate metabolism, vascular biology, inflammation, cell proliferation, and senescence [65,66,67]. To be transcriptionally active, PPARs must heterodimerize with the 9-cis retinoic acid receptor (RXR) (Figure 3) [68]. If an agonist is absent or in the presence of an antagonist, the PPAR-RXR heterodimer associates with co-repressor proteins. This complex occupies the promoter region within a subset of PPAR target genes, and consequently blocks their transcription. Such co-repressor proteins include the well-known silencing mediator of retinoid and thyroid receptors (SMRT), and the nuclear receptor corepressor (NCoR) [68,69,70]. 

On the other hand, in the presence of an agonist, PPAR activation results in an exchange within the co-regulator complex. This involves co-activator recruitment upon co-repressor dissociation. Activated PPAR-RXR heterodimers bind to peroxisome proliferator response elements (PPREs) located in the regulatory regions (5′-end region and introns) of PPAR target genes [68,71,72]. This results in altered expression levels of PPAR target genes. PPAR and RXR bind to the 5′ and 3′ half-sites of the PPRE, respectively [73]. The 5′ flanking region of the PPRE contributes to the selectivity of binding of the different PPAR isotypes [74], but the selection of the PPAR target genes to be activated by a given PPAR isotype in vivo is not yet well understood. It is thought that it results from a complex interplay between expression levels of the three isotypes in the cell, ligand and cofactor availability, affinity for a given PPRE, and probably factors binding in the vicinity of the PPRE [72]. Comprehensive studies integrating expression profiling and genome-wide promoter binding by the PPARs are required to better understand the promoter-specific mechanisms of PPAR action. Interestingly, PPAR/RXR heterodimers can induce transcription in response to PPAR or RXR ligand-dependent activation and the relative levels of cofactor expression are important determinants of the specificity of the physiological responses to PPAR or RXR agonists [72]. Studies of PPARs’ roles in reducing the expression of a subset of inflammatory response genes have highlighted a repressive molecular mode of action, termed transrepression, through which PPARs impact key transcription factor activity. Transrepression occurs through tethering, in which direct protein–protein interactions inhibit the binding of transcription factors to DNA. The regulation of gene transcription by PPAR can also take place through the sequestration of coactivators or the release of corepressors, which stimulates and represses promoter activity, respectively (Figure 3) [72].

The PPAR family includes three isotypes—PPARα, PPARβ/δ, and PPARγ—which have the canonical nuclear receptor domain organization [68,75]. The N-terminal A/B domain possesses a weak ligand-independent transactivation function known as activation function (AF)-1. The C domain binds DNA via two zinc-finger motifs, and the D domain is a hinge region. The E domain contains the ligand-binding domain (LBD), possesses the ligand-dependent transactivation function termed AF-2, and includes the region for dimerization and interaction with regulatory proteins [76,77]. PPARβ/δ also functions in the regulation of gene expression independently of DNA binding, through cross-talk with other transcription factors, which consequently influences their transrepressor function. For example, PPARβ/δ associates with the transcriptional repressor B-cell lymphoma-6 (BCL-6) (Figure 3) in macrophages, endothelial cells, and vascular smooth muscle cells [78,79]. In the presence of a PPARβ/δ agonist, BCL-6 dissociates from PPARβ/δ and subsequently binds to promoter regions of pro-inflammatory genes, such as vascular cell adhesion molecule-1 *(VCAM-1*) and E-selectin. With the aid of a co-repressor complex, such binding will repress the transcription of these genes [29,80,81].

## 4. Hepatic Functions of PPARβ/δ Compared to PPARα and PPARγ

As mentioned above, *Pparα*, *Pparβ/δ*, and *Pparγ* encode proteins with a highly conserved structure and molecular mode of action. However, the receptors differ in their tissue distribution patterns and target genes and, therefore, in the biological functions that they regulate. Below, we briefly review the roles of PPARα and PPARγ, and then discuss those of PPARβ/δ in greater detail.

### 4.1. PPARα

PPARα is predominantly expressed in tissues with high levels of fatty acid catabolism, including the liver, as well as brown adipose tissue, heart, kidney, and skeletal muscle [82,83,84]. In the liver, PPARα is involved in fatty acid metabolism through transcriptional upregulation of numerous genes that play roles in mitochondrial and peroxisomal fatty acid oxidation, and in phospholipid remodeling [85,86,87]. PPARα also participates in downregulating hepatic inflammatory processes by reducing the effects of acute exposure to cytokines [88,89,90,91].

Preclinical and clinical studies have demonstrated that PPARα can influence NAFLD and NASH development [92,93,94,95,96,97]. Fasting is sufficient to trigger steatosis in PPARα-null mice, indicating that PPARα activity is required for metabolizing free fatty acids released from adipocytes [98,99]. Since PPARα is expressed and active in many organs, it is possible that the absence of PPARα in these organs might contribute to the development of fasting-induced steatosis. Therefore, we generated a hepatocyte-specific *Pparα*-null mouse and found that hepatocyte-restricted *Pparα* deletion is sufficient to promote steatosis [97]. This mouse shows impaired whole-body fatty acid homeostasis not only during fasting, but also when fed a methionine- and choline-deficient diet or a high-fat diet. Collectively, these data establish PPARα as a relevant drug target in NAFLD [97].

### 4.2. PPARγ

The PPARγ protein has two isoforms: PPARγ1 and PPARγ2. Differential promoter usage and alternate splicing of the PPARγ gene products actually generate three messenger RNAs (mRNAs)—PPARγ1, PPARγ2, and PPARγ3—with the PPARγ1 and PPARγ3 mRNAs both encoding the PPARγ1 protein [100]. PPARγ isoforms γ1 and γ2 are highly expressed in white and brown adipose tissues, where the receptor governs adipocyte differentiation and lipid storage. PPARγ1 is also expressed in the brain, vascular cells, colon, and immune cells [82,83].

PPARγ is weakly expressed in healthy liver, and steatosis is associated with increased hepatic expression of the PPARγ2 isoform, as observed in various mouse models of obesity [101,102]. Accordingly, hepatocyte-specific PPARγ deletion reduces hepatic fat content in mice fed a high-fat diet [103]. Increased PPARγ2 gene expression is also positively correlated with liver steatosis in obese patients [104,105]. Findings in the hepatocyte-specific PPARγ-knockout model indicated that PPARγ directly promotes hepatic fat accumulation by increasing lipid uptake, and by promoting *de novo* lipogenesis [106,107,108,109,110]. More recently, observations in an original mouse model of inducible hepatocyte-specific PPARγ deletion have suggested that PPARγ plays a specific role in fatty acid uptake and diacylglycerol (DAG) synthesis via upregulation of *Cd36* and monoacylglycerol O-acyltransferase 1 (*Mogat1*) [111]. Moreover, PPARγ plays important roles in glucose metabolism by regulating the expression of hexokinase 2 (HK2) and the M2 isoform of pyruvate kinase (PKM2), resulting in massive liver steatosis in phosphatase and tensin homologs deleted on chromosome 10 (PTEN)-null mice [112].

### 4.3. PPARβ/δ

PPARβ/δ is ubiquitously expressed, with the expression level varying among organs, cells, and species. Hepatic expression is low to moderate in adult humans and rats [82,113,114,115,116] and moderate to high in mice [117]. Pparβ/δ is highly expressed in hepatocytes, liver sinusoidal endothelial cells (LSECs), and liver-resident macrophages (Kupffer cells) [118]. Pparβ/δ expression is also constitutively high in hepatic stellate cells (HSCs).

In liver tissue of *Pparβ/δ*-null mice, transcriptional profiling revealed downregulation of genes associated with lipoprotein metabolism and glucose utilization pathways, indicating that these genes are positively regulated by PPARβ/δ. On the other hand, genes involved in innate immunity and inflammation were upregulated, suggesting their repression by PPARβ/δ. These transcriptional changes in *Pparβ/δ*-null mice correlated with increased plasma glucose and triglyceride levels, and reduced plasma cholesterol levels [119]. These results suggested important roles of PPARβ/δ in energy metabolism and inflammation, which we discuss below.

#### 4.3.1. PPARβ/δ Roles in Energy Metabolism

In a very informative piece of work, Liu et al. demonstrated that adenovirus-mediated liver-restricted PPARβ/δ overexpression reduced fasting glucose levels in both chow- and high fat-fed mice. In parallel an increased hepatic glycogen and lipid deposition was observed accompanied by an up-regulation of glucose utilization and de novo lipogenesis [28]. PPARβ/δ increased the production of monounsaturated fatty acids (MUFAs), which activate PPARs, while reducing saturated fatty acid levels. Lipid accumulation in the adeno-PPARβ/δ-infected livers reduced cell damage and c-Jun N-terminal kinase (JNK) stress signaling. The authors proposed that the PPARβ/δ-regulated lipogenic program may protect against lipotoxicity, and that altered substrate utilization by PPARβ/δ resulted in AMP-activated protein kinase activation, which may contribute to the glucose-lowering activity of PPARβ/δ. Taken together, this data suggested that PPARβ/δ impacts hepatic energy substrate homeostasis by a coordinated control of fatty acid and glucose metabolism [28].

In line with these findings, PPARβ/δ regulates lipogenic genes during the dark/feeding cycle. Specifically, PPARβ/δ drives MUFA production via stearoyl-CoA desaturase 1 (*Scd1*) upregulation, a process that avoids lipotoxicity by increasing fatty acid oxidation or sequestration of saturated fatty acids. As such, the process inhibits saturated fatty acid-induced cytotoxicity in hepatocytes. Furthermore, long chain acyl-CoA from MUFA production allows esterification into triglycerides [120]. Interestingly, liver-specific PPARβ/δ activation increases fatty acid uptake in muscle, whereas its deletion has an opposite effect. Phosphatidylcholine 18:0/18:1 (PC (18:0/18:1)) was identified as a serum lipid produced in the liver under the control of PPARβ/δ activity, which upon circulating to muscles stimulates fatty acid catabolism through PPARα activation [121]. 

For a direct comparison of the roles of *Pparα* and *Pparβ/δ* in liver, microarray analysis was being used to compare the liver transcriptome between *Pparα* and *Pparβ/δ*-null mice, revealing a small overlap in the regulation of genes that are both PPARα- and PPARβ/δ-dependent. In the fed state, similar numbers of genes exhibited altered expression in *Pparα* and *Pparβ/δ* deletion. However, during fasting, more genes showed altered expression in *Pparα*-deleted mice compared to *Pparβ/δ*-null mice. Analysis of plasma metabolites, including free fatty acids and β-hydroxybutyrate, supported the notion that PPARα is particularly important during fasting, while PPARβ/δ appears to be important in both the fed and fasted states [119]. Based on functional similarities to PPARα, PPARβ/δ may be a master regulator of hepatic intermediary metabolism. In rodents, both receptors play non-redundant roles in the liver to enhance ketogenesis through induction of *Fgf21* and expression of fatty acid oxidation genes under fasting conditions [122,123]. In fact, PPARα is an important activator of hepatic fatty acid oxidation [97,99,124]. Interestingly, PPARβ/δ cannot compensate for PPARα in *Pparα*-null mice [98].

The differences between PPARα and PPARβ/δ in molecular and biological functions also corresponded with their antiphasic circadian expression profiles. Indeed, PPARα peaks at the end the light/resting period, while PPARβ/δ is highly expressed in the liver during the night/feeding period, according to [86,121], and Montagner et al., unpublished results. Notably, during fasting (usually light period), PPARβ/δ expression decreases while PPARα is highly expressed [125]. In spite of their biphasic expression profile, intra- and inter-organ dialogs between PPARβ/δ and PPARα activities have been described. As mentioned above, increased hepatic PPARβ/δ activity can lead to PPARα activation in muscle tissue via production of the specific PPARα ligand 16:0/18:1-phosphatidylcholine [121]. This mechanism could also occur in the liver [121,126]. Overall, while both PPARα and PPARβ/δ are associated with the regulation of hepatic lipid metabolism [127,128], hepatic PPARβ/δ mainly acts on anabolic metabolic processes and primarily contributes to glucose utilization, MUFA formation, and anti-inflammatory responses [119,129]. 

Compared with PPARα and PPARγ, less is known about PPARβ/δ in relation to obesity and NAFLD [130]. However, the lipogenic activity of PPARβ/δ raises the question of whether PPARβ/δ activation is associated with steatosis and steatohepatitis. It was recently shown that both PPARβ/δ and PPAR*α* receptors were necessary for adipose tissue reduction driven by the PPARβ/δ agonist GW501516 and subsequent development of hepatic steatosis, with PPARβ/δ working upstream of PPARα [131]. PPARβ/δ is also involved in transforming potentially toxic lipids into less toxic molecules by regulating MUFA synthesis, a process that increases PPARα activity and could protect against NAFLD and promote detoxification. In mice with adenovirus-mediated liver-restricted PPARβ/δ overexpression, examination revealed elevated liver expression of the adiponectin receptor 2 (AdipoR2), leading to enhanced 5′ adenosine monophosphate-activated protein kinase (AMPK) activity [132]. This PPARβ/δ-dependent increase in AMPK activity reportedly suppressed lipogenesis and glycogen synthesis, reduced gluconeogenesis, and increased fatty acid oxidation [25,26,27]. The AMPK pathway may act as a negative feedback loop for PPARβ/δ, possibly explaining why long-term PPARβ/δ agonist treatment does not lead to liver lipid accumulation [133]. Similarly, PPARβ/δ suppresses lipogenesis by lowering SREBP1c levels, reducing the severity of hepatic steatosis in obese diabetic *db/db* mice via stimulation of the insulin-induced gene-1 (*Insig-1*), the product of which inhibits SREBP1c [134]. 

Fibroblast growth factor 21 (FGF21) is a circulating hormone derived from the liver, which plays important roles in regulating glucose and lipid metabolism [135,136]. Recent evidence shows that PPARβ/δ and FGF21 exert hepatic regulation of the VLDL receptor, which modulates NAFLD. Liver tissue of *Pparβ/δ*-null mice and *Pparβ/δ−/−* hepatocytes exhibit increased VLDL receptor expression. Moreover, FGF21 neutralizing antibody treatment resulted in triglyceride accumulation in *Pparβ/δ*-null mice [137]. In support of these pre-clinical results, liver biopsies from patients with moderate and severe hepatic steatosis showed increased VLDL receptor levels and reduced PPARβ/δ mRNA levels and DNA-binding activity compared to in control subjects. These findings revealed a novel mechanism in which VLDL receptor levels are controlled by PPARβ/δ and FGF21, impacting hepatic steatosis development [137].

#### 4.3.2. PPARβ/δ Roles in Inflammation

On a high-fat diet, the PPARβ/δ-dependent increase in hepatocyte MUFA production impacts liver-resident macrophages and Kupffer cells—resulting in increased PPARβ/δ activation, and reduced expression of TNFα or interferon gamma (IFNγ) inflammatory markers from these cells—and altering the immune response [28]. Thus, this finding suggests that PPARβ/δ plays an anti-inflammatory role in liver. PPARβ/δ and its ligands are also reportedly associated with anti-inflammatory activities through interference with nuclear factor kappa-light-chain-enhancer of activated B cells (NFκB) signaling [67,138,139] and through interactions with signal transducer and activator of transcription 3 (STAT3) and extracellular-signal-regulated kinase 5 (ERK5) [140,141]. 

Kupffer cells are also involved in insulin resistance and fatty liver disease [142], and PPARβ/δ plays a role in regulating the alternative activation of these cells [143]. In the presence of IL4 and IL13 stimulation, PPARβ/δ is required for the activation of Kupffer cells to the M2 subtype that has anti-inflammatory activity. Hematopoietic *Pparβ/δ*-deficient obese mice exhibited lower insulin sensitivity and oxidative metabolism, as well as impaired alternative activation of Kupffer cells. This phenotype was validated by three independent lines of experiments. First, *Pparβ/δ* deletion in lean mice resulted in lower expression of genes involved in alternatively activated Kupffer cells, such as arginase 1 (*Arg1*), c-type lectin domain containing 7A (*Clec7a*), jagged 1 (*Jag1*), programmed cell death 1 ligand 2 (*Pdcd1lg2*) and chitinase (*Chia*). However, treatment with PPARβ/δ agonist GW0742 led to increased expression of these genes in liver. Second, replacing the bone marrow of wild-type mice with *Pparβ/δ*-null bone marrow led to insulin resistance and mitochondrial dysfunction in hepatocytes, eliminating the alternative activation of Kupffer cells. Third, direct co-culturing of *Pparβ/δ*-null macrophages with primary hepatocytes induced a significant reduction of oxidative phosphorylation in the parenchymal cells. The study demonstrated the association between *Pparβ/δ*-null Kupffer cells and dysregulation of hepatic metabolism, resulting in increased liver triglycerides [143]. 

PPARβ/δ is also involved in hepatic stellate cell (HSC) activation; its expression is upregulated in cultures of activated HSCs and in in vivo fibrogenesis [144,145]. Administration of the PPARβ/δ agonist L165041 enhances HSC proliferation, and L165041 administration combined with chronic carbon tetrachloride (CCl_4_) treatment leads to higher fibrotic marker expression in rats [146]. These data suggested that PPARβ/δ plays an important role as a signal-transducing factor, leading to HSC proliferation in the event of acute and chronic liver inflammation [146]. In activated HSCs, PPARβ/δ enhances the expression of *Cd36*, which codes for a membrane receptor that facilitates fatty acid uptake. Moreover, upregulated PPARβ/δ expression is associated with elevated expression of proteins involved in retinoid binding and esterification, such as cellular retinol-binding protein 1 (CRBP-1) and lecithin retinol acyltransferase (LRAT). Overall, PPARβ/δ regulates the expression of genes related to vitamin A metabolism in HSCs undergoing activation [144].

Interestingly, CCl_4_-induced hepatic fibrotic response requires PPARβ/δ which enhances expression of profibrotic and pro-inflammatory genes in mice. This process results in increased macrophage recruitment and extracellular matrix deposition in the liver [145]. However, this phenotype was not observed in *Pparβ/δ*-null mice treated with CCl_4_ alone or with CCl_4_ plus GW501516. The same study further demonstrated that GW501516 administration increased HSC proliferation in CCl_4_-injured wild-type mice livers, but not in *Pparβ/δ*-null mice with the same treatment. In another study, GW501516-treated *db/db* mice exhibited higher expression of the lipogenic enzyme acetyl-CoA carboxylase β and elevated triglyceride levels in the liver [147]. Moreover, investigations of GW501516 treatment in control and *Pparβ/δ*-knockdown LX-2 human hepatic stellate cells revealed that GW501516-stimulated HSC proliferation occurs via p38 and JNK mitogen-activated protein kinase (MAPK) pathways [145]. However, in the same model of CCl_4_-induced liver damage, administration of the PPARβ/δ agonist KD3010 (chemical abstracts service, CAS ID 934760-90-4) ameliorated the CCl_4_-induced liver injury with lower deposition of extracellular matrix proteins. KD3010 treatment of primary hepatocytes provided protection from CCl_4_-induced cell death or starvation, suggesting that KD3010 administration could have hepatoprotective and antifibrotic effects in animal models of liver fibrosis [148]. Further studies are needed to determine the reasons for the different effects of GW501516 and KD3010 in injured livers [149].

In mice treated with the agonist GW0742, NFκB signaling was attenuated in a PPARβ/δ-dependent manner. Compared to wild-type mice, *Pparβ/δ*-null mice exhibited higher TNFα and αSMA expression in hepatocytes and HSCs, but similar inflammatory signaling in hepatocytes and activation of HSCs [150]. A recent study using the same PPARβ/δ agonist demonstrated that PPARβ/δ upregulates serum high-density lipoprotein (HDL) and HDL phospholipids in NAFLD mice, while this effect is not seen in *Pparβ/δ*-deficient mice [151].

## 5. Pharmacological Strategies Targeting PPARβ/δ for NAFLD Treatment

### 5.1. PPARβ/δ Agonists: GW0742, GW501516

Preclinical studies have investigated long-term treatment with PPARβ/δ agonists such as GW0742 (CAS ID 317318-84-6) and GW501516 (CAS ID 317318-70-0) in animal models, revealing that PPARβ/δ activation attenuates hepatic steatosis by promoting fatty acid oxidation, reducing lipogenesis, and enhancing insulin sensitivity [134,152,153,154]. On the contrary, short-term treatment with PPARβ/δ agonists reportedly yields a transient increase in hepatic triglyceride levels [131]. Elevated levels of monounsaturated fatty acids, are accompanied by lower saturated fatty acid levels and no observed hepatotoxicity [28]. Studies involving PPARβ/δ agonist treatment in humans have demonstrated reduced hepatic fat content and improved plasma markers of liver function, including carnitine palmitoyltransferase 1b [155,156]. One study conducted in middle-overweight patients revealed that GW501516 treatment decreased liver lipid content and insulinemia, with no signs of oxidative stress [156]. However, LDL cholesterol plasma level was also reduced. This suggests that the protective effects of PPARβ/δ pharmacological activation are reliant on increased lipid oxidation in muscles. 

### 5.2. PPAR Dual Agonists: Elafibranor, Saroglitazar

The PPARα and PPARβ/δ dual agonist elafibranor (also known as GTF-505, CAS ID 923978-27-2) has recently emerged as one of the most promising chemical entities for treatment of NAFLD, especially NASH. Prior studies have demonstrated its efficiency, and it is currently undergoing phase III testing in NASH patients. It has reportedly improved steatosis, inflammation, and fibrosis in mouse models of NAFLD [95], and thus appears to be a good candidate for the treatment of hepatic fibrosis, NAFLD, primary biliary cirrhosis, and NASH. Elafibranor was investigated in a randomized, double-blind, placebo-controlled trial including 274 patients in Europe and the USA (GOLDEN-505 trial; NCT01694849). Post-hoc analysis of those trial results revealed that ALT was significantly reduced after four to 12 weeks of elafibranor treatment among patients who were in the top two quartiles at baseline. Non-cirrhotic patients with NASH did not exhibit any worsening of hepatic fibrosis after 52 weeks of taking elafibranor at 120 mg/day [157]. Liver biopsy analysis in this patient group further revealed disappearance of hepatocellular ballooning, with no or mild lobular inflammation. Elafibranor-treated patients also exhibited improvement in liver enzymes, lipid parameters (triglycerides, low-density lipoprotein, high-density lipoprotein, and cholesterol), serum inflammation biomarkers, steatosis, and fibrosis. Other studies have reported that elafibranor treatment improves glucose homeostasis and insulin resistance in diabetic patients [157,158]. Overall, elafibranor appears to be safe and well-tolerated, with no deaths or cardiovascular incidents reported during treatment. There is currently an ongoing phase III randomized, double-blind, placebo-controlled trial of elafibranor use in 2000 liver biopsy-proven NASH patients, to investigate the efficacy against NASH and the safety regarding fibrosis during longer use (72 weeks) (NCT02704403) [159].

Interestingly, the PPARα/γ dual agonist saroglitazar (CAS ID 495399-09-2) has also exhibited overall beneficial effects in experimental models of NASH [160]. Moreover, saroglitazar treatment induces a significant decrease of ALT levels in subjects with biopsy-proven NASH [21]. Since saroglitazar improves all of the components responsible for NAFLD/NASH in preclinical models, it is also a promising candidate for the management of these conditions. Further studies are needed to examine the possible common and different pathways of action of elafibranor and saroglitazar.

### 5.3. PPAR Pan-Agonists: Bezafibrate, MHY2013, Lanifibranor

The anti-fibrotic and anti-inflammatory effects of PPARs have inspired growing use of PPAR pan-agonists to treat NAFLD. It is postulated that PPAR pan-agonist may show improved efficacy compared to targeting a single PPAR isotype [161]. The PPAR pan-agonist bezafibrate (CAS ID 41859-67-0), which activates PPARα, PPARβ/δ, and PPARγ, has shown beneficial effects in NASH treatment. In mice fed a methionine- and choline-deficient diet, bezafibrate and GW501516 (selective PPARβ/δ agonist) treatments have resulted in upregulation of β-oxidation and lipid transport genes in hepatocytes. They have inhibited NASH development. These treatments also both resulted in reduced inflammatory gene expression [152]. MHY2013 is another PPAR pan-agonist that also activates all three PPAR isotypes. In aged Sprague-Dawley (SD) rats, MHY2013 treatment improved age-related hepatic lipid accumulation, and resulted in upregulated β-oxidation signaling and lower inflammation in the liver [162]. The PPAR pan-agonist Lanifibranor (CAS ID 927961-18-0) is reportedly effective in experimental skin and lung fibrosis [163,164]. It has been proposed for use as an anti-fibrotic treatment. Lanifibranor is currently being tested in a phase 2b randomized, double-blind, placebo-controlled trial for safety and efficacy in up to 225 patients in 12 European countries (NCT03008070) [165].

## 6. Conclusions

NAFLD is an alarming health issue that is occurring with rising frequency in developed countries. It is now well documented that PPARβ/δ is involved in regulating glucose and lipid metabolism in the liver. An improved understanding of the physiological roles of PPARs, particularly PPARβ/δ, will likely contribute to the design and development of safe agonists with enhanced therapeutic potential compared to first-generation agonists. Although much remains unknown about the physiological impact of PPARβ/δ, prior research has elucidated highly interesting NAFLD-related functions, as reviewed in this article. 

Some results on PPARβ/δ roles seem contradictory, and the reasons for these discrepancies is unclear. It is conceivable that PPARβ/δ exert different functions in a context- and agonist-specific manner. For example, one study reported that PPARβ/δ stimulates the *de novo* lipogenesis pathway, which is accompanied by lipid deposition. Interestingly, this PPARβ/δ-regulated lipogenic program is paralleled by reduced JNK stress signaling, suggesting that it may protect against lipotoxicity [28]. However, it has also been suggested that PPARβ/δ suppresses hepatic lipogenesis. PPARβ/δ overexpression enhanced *Insig-1* expression, which suppressed SREBP-1 activation and thus ameliorated hepatic steatosis in obese *db/db* mice [134]. Similarly, PPARβ/δ agonists GW501516 and KD3010 exerted pro-fibrotic and anti-fibrotic effects, respectively, in CCl_4_-injured livers [145,146]. Uncovering the causes for these apparent discrepancies will likely elucidate differentiated responses of PPARβ/δ in specific situations, which will be important for PPARβ/δ as a pharmacological target. We are in the opinion that detail transcriptomic profiling in combination with a better understanding of the pharmacological characteristics of candidate drugs, such as half-life, affinity constant, and bioavailability, may provide insights into their true target and reveal potential off-target effects.

PPARβ/δ also plays an interesting role in the alternative activation of Kupffer cells to the anti-inflammatory macrophage M2 subtype [143], revealing the direct PPARβ/δ-dependent involvement of Kupffer cells in liver lipid metabolism. Based on this beneficial role for alternatively activated Kupffer cells in metabolic syndrome conditions, controlling PPARβ/δ activity in these cells may contribute to delaying NAFLD progression.

The fine tuning of PPAR-regulated physiological functions in the liver and other organs is influenced by the functional interaction between PPARβ/δ and PPARα [121,131]. PPARβ/δ apparently works upstream of PPARα, controlling the production of MUFAs, as well as PC (18:0/18:1), which activates muscle PPARα to increase muscle energy use [121]. MUFAs also activate PPARα in the liver itself. This regulatory circuit couples ligand production and the activities of two receptors that play key roles in liver energy metabolism.

**Figure 4:**
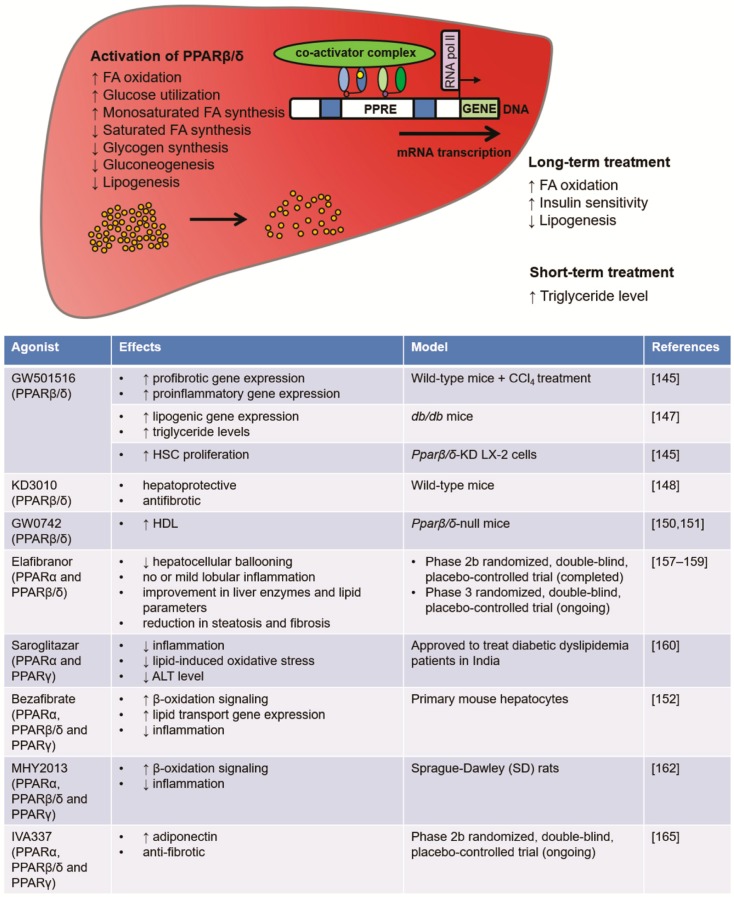
Overview of research findings regarding PPARβ/δ in hepatic metabolism, and the contrasting effects of various PPARβ/δ agonist treatments in pre-clinical models.

These complex interactions are certainly of interest for the development of novel PPAR drugs. PPARα/PPARβ/δ dual agonists may have additional beneficial effects due to the integrated roles of these two receptors through the abovementioned regulatory circuit they form together. GFT505 (elafibranor) is the most advanced PPARα/PPARβ/δ dual agonist [158]. It has been tested in several clinical trials and is currently being evaluated in a clinical phase III study [166]. Several other PPAR agonists, dual agonists, and pan-agonists of interest have been investigated, and some are now in clinical studies of safety and efficacy (Figure 4). As PPARs play important roles in regulating genes involved in fatty acid uptake and oxidation [65,66,67], we propose that targeting PPARs will be one of the best possibilities to treat fatty liver diseases.

## Abbreviations

ADIPOR2	adiponectin receptor 2
AF	activation function
APOB	apolipoprotein B
Arg1	arginase 1
CAS	chemical abstracts service
CCL_4_	chronic carbon tetrachloride
CHIA	chitinase
CLEC7a	c-type lectin comain containing 7A
DAG	diacylglycerol
DBD	DNA-binding domain
ERK5	extracellular-signal-regulated kinase 5
FABP	fatty acid-binding protein
FGF21	fibroblast growth factor 21
GCKR	glucokinase regulator
GWAS	genome-wide association studies
HCC	hepatocellular carcinoma
HK2	hexokinase 2
HSC	hepatic stellate cell
IFNγ	interferon gamma
IL	interleukin
INSIG-1	insulin-induced gene-1
JAG1	jagged 1
JNK	c-Jun N-terminal kinase
LBD	ligand-binding domain
LDL	low-density lipoprotein
LSEC	liver sinusoidal endothelial cell
LYPLAL1	lysophospholipase like 1
MOGAT1	monoacylglycerol O-acyltransferase 1
MUFA	monounsaturated fatty acids
NAFLD	non-alcoholic fatty liver disease
NASH	non-alcoholic steatohepatitis
NCAN	neurocan
NCoR	nuclear receptor corepressor
NFκB	nuclear factor kappa-light-chain-enhancer of activated B cells
NTD	N-terminal domain
PC	Phosphatidylcholine
Pdcd1lg2	programmed cell death 1 ligand 2
PKM2	M2 isoform of pyruvate kinase
PNAPL3	patatin-like phospholipase domain containing 3
PPAR	peroxisome proliferator-activated receptor
PPP1R3B	protein phosphatase 1 regulatory subunit 3B
PPRE	peroxisome proliferator response element
PTEN	phosphatase and tensin homolog deleted on chromosome 10
RXR	retinoic acid receptor
SCD1	stearoyl-CoA desaturase 1
SMRT	silencing mediator of retinoid and thyroid receptors
SREBP1c	sterol-regulated binding protein 1c
STAT3	signal transducer and activator of transcription 3
TF	transcription factor
TFBS	TF-binding site
TM6SF2	transmembrane 6 superfamily member 2
TNFα	tumor necrosis factor α
TZD	thiazolidinedione
VCAM-1	vascular cell adhesion molecule-1
VLDL	very low-density lipoprotein
